# Successful surgical management of non-perforating acute appendicitis with septic disseminated intravascular coagulation: A case report and review of the literature

**DOI:** 10.1016/j.ijscr.2019.01.016

**Published:** 2019-01-29

**Authors:** Toshiaki Komo, Toshihiko Kohashi, Yoshirou Aoki, Jun Hihara, Koichi Oishi, Noriaki Tokumoto, Mikihiro Kanou, Akira Nakashima, Manabu Shimomura, Masashi Miguchi, Hidenori Mukaida, Naoki Hirabayashi

**Affiliations:** a Department of Gastroenterological Surgery, Hiroshima City Asa Citizens Hospital, Japan; b Department of Gastroenterological and Transplant Surgery, Applied Life Sciences, Institute Biomedical & Health Sciences, Hiroshima University, Japan

**Keywords:** DIC, disseminated intravascular coagulation, CT, Computed tomography, Non-perforating appendicitis, Uncomplicated appendicitis, Septic, Disseminated intravascular coagulation (DIC)

## Abstract

**Introduction:**

Perforating appendicitis and abscess-forming appendicitis may cause septic disseminated intravascular coagulation (DIC). However, non-perforating acute appendicitis with septic DIC is extremely rare.

**Presentation of case:**

A 67-year-old man was referred to our hospital one day after starting oral antibiotic treatment for acute appendicitis. Physical examination revealed only slight spontaneous abdominal pain without tenderness and peritoneal irritation. Contrast-enhanced computed tomography demonstrated an enlarged appendix (10 mm in diameter) without fecalith, ascites, intraperitoneal free air, and abscess. There was no evidence of perforating appendicitis. Laboratory analysis revealed septic DIC. The patient was diagnosed with non-perforating acute appendicitis with septic DIC. The patient was distressed regarding whether he should be treated conservatively with an antibiotics-first strategy or undergo an appendectomy. Ultimately, a laparoscopic appendectomy was performed. Histopathological examination showed non-perforating gangrenous appendicitis. He required DIC therapy for 2 days postoperatively. He was discharged on postoperative day 9, and remained in good health 1 month after surgery.

**Discussion:**

There is no absolute index of conversion to surgery with an antibiotics-first strategy of appendicitis treatment. Judging the limit of conservative treatment and determining the best moment to perform surgery is a critically important matter for patients with acute appendicitis.

**Conclusions:**

The incidence of conservative treatment preceding an antibiotics-first strategy for acute appendicitis is increasing. However, it is thought that appendectomy should be performed when acute appendicitis is complicated with septic DIC, even if it is a non-perforating appendicitis in which improvement with conservative treatment is anticipated.

## Highlights

• Non-perforating acute appendicitis with septic DIC is extremely rare.
• Nowadays, interval appendectomy has become popular.
• However, judging the limit of conservative treatment for acute appendicitis is a critically important matter.
• Appendectomy should be performed for non-perforating acute appendicitis with septic DIC.

## 1 Introduction

Acute appendicitis is one of the most common gastrointestinal emergencies. Appendectomy is still considered the gold standard for acute appendicitis treatment. Nowadays, interval appendectomy is often used [1]. Many cases are relieved by conservative treatment, even those with perforating appendicitis without peritoneal irritation and abscess in the abdominal cavity. Needless to say, it is very important not to miss the time point when conservative treatment should stop and conversion to surgery should occur.

Generally, perforating appendicitis and abscess-forming appendicitis may cause septic disseminated intravascular coagulation (DIC). However, it is difficult to assume that acute appendicitis lacking abdominal symptoms and without perforation and abscess may cause septic DIC. We report herein a rare case of non-perforating acute appendicitis with septic DIC. The present work has been reported in accordance with the SCARE criteria [2].

## 2 Case presentation

**Fig. 1:**
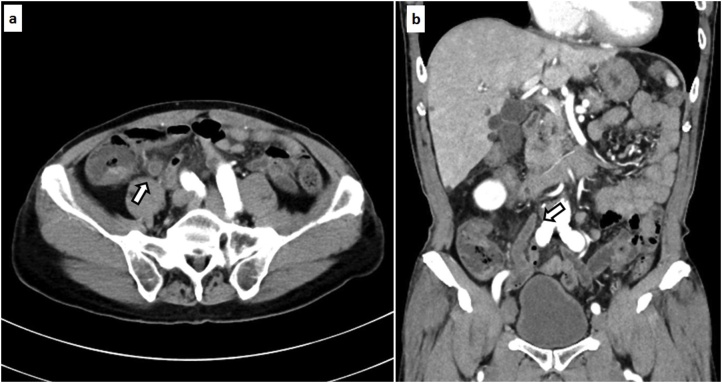
Contrast-enhanced CT demonstrated an enlarged appendix (10 mm in diameter) without fecalith, ascites, intraperitoneal free air, and abscess (appendix: thick white arrow). a) Axicial view, b) Coronal view.

**Fig. 2:**
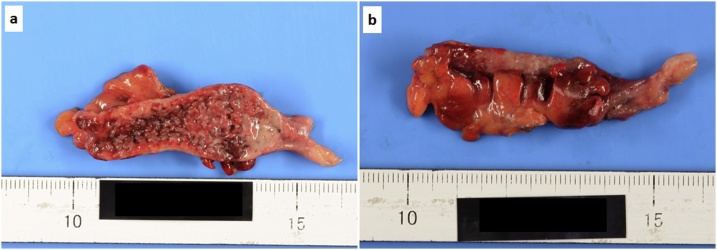
The resected specimen revealed a necrotized appendiceal mucous membrane. There was no evidence of appendiceal wall perforation. a) Mucous membrane side, b) Serosa side.

A 67-year-old man with no medical history consulted a nearby doctor for the main complaints of fever and lower abdominal pain. Laboratory analysis revealed hemoglobin, 13.1 g/dL; white blood cell count, 13.76 × 10^3^/μL; platelets, 12.7 × 10^4^/μL; and C-reactive protein, 1.41 mg/dL. He was diagnosed with acute appendicitis, and oral antibiotic treatment was initiated. On the following day, he was referred to our hospital for suspected DIC, as laboratory analysis revealed hemoglobin, 13.3 g/dL; white blood cell count, 3.55 × 10^3^/μL; platelets, 7.4 × 10^4^/μL; and C-reactive protein, 12.2 mg/dL. At the time of hospital consultation, physical examination revealed stable cardiorespiratory dynamics and a fever of 38.3 °C, no abdominal distension, and only slight spontaneous abdominal pain without tenderness and peritoneal irritation. Laboratory analysis revealed hemoglobin, 14.0 g/dL; white blood cell count, 9.41 × 10^3^/μL; platelets, 6.9 × 10^4^/μL; serum total protein, 5.2 g/dL; serum albumin, 3.3 g/dL; total bilirubin, 1.6 mg/dL; aspartate aminotransferase, 218 IU/L; alanine aminotransferase, 198 IU/L; lactic acid dehydrogenase, 315 IU/L; blood urea nitrogen, 20 mg/dL; creatinine, 0.96 mg/dL; C-reactive protein, 13.47 mg/dL; prothrombin activation, 54%; international normalized ratio of prothrombin time, 1.36; fibrinogen/fibrin degradation products, 116.4 μg/mL; and antithrombin III activity, 70%. The sequential organ failure assessment score was 2 points. The Japanese Association for Acute Medicine DIC diagnostic criteria score [3] was 7 points (platelet counts; 3 points, prothrombin time; 1 point, and fibrin/fibrinogen degradation products; 3 points). Contrast-enhanced computed tomography (CT) demonstrated an enlarged appendix (10 mm in diameter) without fecalith, ascites, intraperitoneal free air, and abscess (Fig. 1). There was no evidence of perforating appendicitis. Laboratory analysis revealed septic DIC. The patient was diagnosed with non-perforating acute appendicitis with septic DIC. The patient was distressed regarding whether he should be treated conservatively with an antibiotics-first strategy or undergo an appendectomy, because he had few symptoms, no perforation, and no abscess. Ultimately, laparoscopic appendectomy was performed due to anxiety about exacerbation of septic DIC. The resected specimen revealed a necrotized appendiceal mucous membrane. There was no evidence of appendiceal wall perforation (Fig. 2). Histopathological examination showed non-perforating gangrenous appendicitis. He required DIC therapy (thrombomodulin administration, antithrombin administration, and nafamostat mesilate) for 2 days postoperatively. Preoperative blood culture detected *Bacteroides thetaiotaomicron*. He was discharged on postoperative day 9, and remained in good health 1 month after surgery.

## 3 Discussion

Acute appendicitis is one of the most common gastrointestinal emergencies. There are two types of appendicitis: non-perforating (uncomplicated) and perforating (complicated) appendicitis. Appendectomy is still considered the gold standard for acute appendicitis treatment. Therefore, it is recommended that appendectomy be performed as soon as possible.

Nowadays, interval appendectomy has become popular as an elective surgical strategy for acute appendicitis following curative antibiotic treatment [1]. Interval appendectomy can avoid extended surgery (cecectomy or ileocecal resection rather than appendectomy alone) and reduce postoperative complications [4]. The indication for interval appendectomy includes patients with perforating appendicitis without general peritonitis and abscess-forming appendicitis, though it is controversial [5]. However, delaying an appendectomy increases the risk of perforating appendicitis with general peritonitis, which is associated with a higher incidence of short and long-term morbidity [6]. Judging the limit of conservative treatment and determining the best time to perform surgery is a critically important matter. Some factors may help to identify the need for a conversion to surgery with an antibiotics-first strategy of appendicitis treatment, such as physical factors (fever, cardiorespiratory, and peritoneal irritation), laboratory parameters (white blood cell count and C-reactive protein), and CT (appendix diameter, ascites, intraperitoneal free air, and abscess). However, there is no absolute index. Therefore, determination of the best time to perform surgery is dependent heavily on the decision of the physician.

In the present case, physical examination revealed stable cardiorespiratory dynamics, no abdominal distension, and only slight spontaneous abdominal pain without tenderness and peritoneal irritation. Contrast-enhanced CT demonstrated an enlarged appendix (10 mm in diameter) without fecalith, ascites, intraperitoneal free air, and abscess (Fig. 1). There was no evidence of perforating appendicitis. Laboratory analysis revealed septic DIC. The patient was diagnosed with non-perforating acute appendicitis with septic DIC. The patient was distressed regarding whether he should be treated conservatively with an antibiotics-first strategy or undergo appendectomy, because he had few symptoms, no perforation, and no abscess. Ultimately, laparoscopic appendectomy was performed due to anxiety about exacerbation of septic DIC.

Histopathological examination showed non-perforating gangrenous appendicitis. It was thought that non-perforating acute appendicitis with septic DIC was caused by the draining of intestinal bacteria from the necrotized appendiceal mucous membrane into the portal venous system due to a rise in the internal pressure of the appendix and necrosis of its mucous membrane. In the present case, preoperative blood culture detected *Bacteroides thetaiotaomicron*. *Bacteroides thetaiotaomicron,* a gram-negative anaerobic bacillus, belongs to the genus *Bacteroide.* Most of the time, *Bacteroides thetaiotaomicron* is part of the normal flora of the gastrointestinal tract, oral cavity, and respiratory tract. The mortality rates of *Bacteroides thetaiotaomicron* sepsis have been reported very high as 17–100% [7]. The patient might not be saved from septic DIC if determination of performing surgery was late.

To the best of our knowledge, non-perforating acute appendicitis with septic DIC is extremely rare, and only a few cases have been reported; only 3 [[8], [9], [10]] and 10 cases [[11], [12], [13], [14], [15], [16], [17], [18], [19], [20]] are reported in the English and Japanese literature, respectively. Fourteen cases, including the present case of non-perforating acute appendicitis with septic DIC, were reviewed in the present study (Table 1). There were 11 (79%) males and 3 (21%) females with a mean age of 50 (range: 26–79) years. Of these 14 patients, 11 did not have peritoneal irritation. There was no evidence of perforating appendicitis with contrast-enhanced CT. The median duration from symptom onset to a diagnosis of septic DIC was 24 (range: 2–48) hours. Nine patients required an estimated 24 h or more for a diagnosis of septic DIC from the onset. Thirteen patients who underwent surgery were alive, and 1 patient who did not was dead. Namely, all 13 of the patients who underwent surgery were saved from septic DIC due to appendectomy; in contrast, 1 patient without appendectomy was not saved from septic DIC. Therefore, appendectomy should be performed for patients with non-perforating acute appendicitis with septic DIC, even if they have few symptoms and no perforation.

**Table 1:** Reported cases of non-perforating acute appendicitis with septic DIC.

No.	Author	Year	Age	Sex	Duration from the onset to a diagnosis of septic DIC	peritoneal irritation	Blood culture	Surgery	Outcome
1	Fredlund [8]	1974	44	F	6 hours	No	Unknown	Yes	Alive
2	Shibata [11]	1981	45	M	24 hours	No	Unknown	No	Dead
3	Pastorek [9]	1982	26	F	36 hours	Yes	E. coli	Yes	Alive
4	Yamazaki [12]	1993	69	M	30 hours	No	Unknown	Yes	Alive
5	Nakamura [13]	2004	61	M	24 hours	Yes	E. coli	Yes	Alive
6	Ito [14]	2005	57	M	Unknown	No	E. coli	Yes	Alive
7	Takeuchi [15]	2006	30	M	36 hours	Yes	Streptococcus	Yes	Alive
8	Nishio [16]	2009	61	M	8 hours	No	Streptococcus	Yes	Alive
9	Hamatsu [17]	2009	34	M	18 hours	No	Unknown	Yes	Alive
10	Rodriguez [10]	2015	43	F	2 hours	No	Unknown	Yes	Alive
11	Yokoyama [18]	2015	39	M	40 hours	No	Peptostreptococcus prevotii	Yes	Alive
12	Sai [19]	2017	76	M	24 hours	No	E. coli	Yes	Alive
13	Higashimoto [20]	2018	35	M	48 hours	No	Eubacuterium	Yes	Alive
14	Our case	2018	79	M	24 hours	No	Bacteroides thetaiotaomicron	Yes	Alive

## 4 Conclusions

We report herein a rare case of non-perforating acute appendicitis with septic DIC. Nowadays, conservative treatment precedes surgery, as non-operative management with an antibiotics-first strategy of acute appendicitis treatment is increasing, even if the patient has perforating appendicitis without general peritonitis and abscess-forming appendicitis. However, it is thought that appendectomy should be performed when acute appendicitis is complicated by septic DIC, even if it is a non-perforating appendicitis in which improvement with conservative treatment is anticipated.

## Conflicts of interest

The authors declare that they have no Conflicts of interest.

## Funding

The authors declare that this study was not funded externally.

## Ethical approval

The study such as this case report was exempted from ethical approval by the Institutional Review Board of Hiroshima City Asa Citizens Hospital.

## Consent

When obtaining informed consent for surgical procedures, general consent for publication and presentation was obtained from the patient.

## Author contribution

TK drafted the manuscript. TK, TK and YA reviewed and edited the manuscript. TK, KO, TK, NT, MK, AN, MS, and MM participated in the care of the patients. MK provided the histopathological examination and diagnosis. YA, JH, TK, MF, HM, and NH participated in critical revision of the manuscript. All authors read and approved the final manuscript.

## Registration of research studies

This is case report not research study.

## Guarantor

Toshihiko Kohashi.

## Provenance and peer review

Not commissioned, externally peer-reviewed.
